# Popularity of Video Games and Collective Memory

**DOI:** 10.3390/e24070860

**Published:** 2022-06-23

**Authors:** Leonardo O. Mendes, Leonardo R. Cunha, Renio S. Mendes

**Affiliations:** Physics Department and National Institute of Science and Technology for Complex Systems, State University of Maringá, Maringá 87020-900, PR, Brazil; leoribeirocunha@gmail.com (L.R.C.); rsmendes@dfi.uem.br (R.S.M.)

**Keywords:** video games, popularity, multiplicative processes, memory effects

## Abstract

Describing the permanence of cultural objects is an important step in understanding societal trends. A relatively novel cultural object is the video game, which is an interactive media, that is, the player is an active contributor to the overall experience. This article aims to investigate video game permanence in collective memory using their popularity as a proxy, employing data based on the *Steam* platform from July 2012 to December 2020. The objectives include characterizing the database; studying the growth of players, games, and game categories; providing a model for the relative popularity distribution; and applying this model in three strata, global, major categories, and among categories. We detected linear growth trends in the number of players and the number of categories, and an exponential trend in the number of games released. Furthermore, we verified that lognormal distributions, emerging from multiplicative processes, provide a first approximation for the popularity in all strata. In addition, we proposed an improvement via Box–Cox transformations with similar parameters (from −0.12 (95% CI: −0.18, −0.07) to −0.04 (95% CI: −0.08, 0)). We were able to justify this improved model by interpreting the magnitude of each Box–Cox parameter as a measure of memory effects.

## 1. Introduction

The concept of memory is ubiquitous both in everyday conversations and in scientific investigations. Our identities, personal, familial, and national, are molded from memories rooted in interpersonal interactions, top-down discourses (e.g., if a nation decides to celebrate one political holiday but not another), and self-reflection. In terms of cultural objects, their popularity at the time when they are made public and in the following years can be seen as a proxy to gauge long-term permanence in collective memory. Thus, one could assume that if a new cultural object was met with a significant and positive reception, it is plausible that such an object will remain longer in collective memory.

This work is dedicated to investigating video game popularity via lognormal distributions and their deformations by Box–Cox transformations. This study is inserted in the broad context of collective and, more specifically, cultural memory. The latter can be characterized, as completed by Assmann [1], as “[…] a form of collective memory in that a number of people share cultural memory and in that it conveys to them a collective (i.e., cultural) identity”. Usually, studies on memory and forgetting of products analyze data stemming from the consumption of books, movies, etc. [2]. This work presents two novelties in this debate. The first is the focus on the popularity of video games over time as indicative of their permanence in collective memory. Multiplicative processes (lognormal distributions) are associated with a memoryless player distribution and deformations of such processes (Box–Cox transformations) with memory effects. The main metrics employed here to quantify how popular video games are is the average number of players of each game per month from July 2012 to December 2020. Other metrics employed as well are the release date and categories of each video game.

The second novelty is associated with the choice of memory studies as a framework for interpreting our quantitative model. While memory studies were already employed in the investigation of the forgetting process for cultural objects [3], this article is the first, to the best of our knowledge, in the context of video game popularity. A valid consideration is in regard to the time frame studied, since cultural memory is usually concerned with intergenerational continuity and longer time intervals. However, in this digital age, new cultural trends are created, appropriated, resignified, and discarded in an almost continuous manner, processes influenced by interpersonal interactions, social networks, and the presence (even pervasiveness) of online advertisement. Thus, in an ever-accelerating society, it seems plausible to discuss cultural objects and memories over shorter time frames and this discussion becomes more relevant in a cultural context dominated by younger people [4,5].

At this point, an objection may be raised: why study video game popularity? To respond to this objection, it should be noted that the socioeconomic role of digital games has been growing, which is reflected in the increased scientific interest from diverse research areas such as education/training [6,7,8,9,10,11], computer/network science [12,13,14,15,16], psychology [17,18,19], and human health/neurology [20,21,22,23]. Of particular interest for the present work, it is worth mentioning that studies are investigating the popularity of video game categories [24,25] and which factors are responsible for keeping players invested in this hobby, the main ones being online play with friends (social factor), intrinsic fun of the game (immersive factor), and achievements (individual factor) [26,27,28,29]. In addition to the increased scientific interest, video games have a value of their own as a cultural object since, unlike most other objects, they are an interactive media, which means that the players are active contributors to the overall experience. In other words, only by understanding user preferences can one fully comprehend video games as cultural phenomena.

The article starts with a data analysis to better understand the dataset, where the growth of the players and games over time was characterized. A model for the relative popularity of video games is proposed, with the simplistic assumption of no prior knowledge of the video game before its release. Afterward, a model that refines this initial assumption is advanced, one that depends on the Box–Cox transformation. Given the natural classification of video games into categories and the existent interest in the literature, the investigation proceeds to consider the relative popularity in the most popular categories using the same models, with the underlying assumption that a model that accurately describes the global layer will be able to reasonably describe its parts. Finally, this work examines the temporal evolution of the number of distinct categories and the probability distribution of all categories according to the number of games in each of them. This examination was guided by the two conjectures. The first is that, while a new video game may grow and decline in popularity over months, it is reasonable to assume that categories are more resilient since they represent the preferences and sensibilities of a fraction of the population. The second is whether the more popular categories are the ones with more options to choose from, i.e., the ones with more games.

From the above discussion, the following conjectures will be explored and investigated in this work:

- The number of video games and of players are growing either linearly or exponentially over time;
- The relative popularity among games is described by a distribution that resembles the lognormal one, a hypothesis which was driven by the fact that this distribution has been employed and studied in the context of econophysics [30], quantitative linguistics [31], and in the popularity analysis of other cultural products, such as patent citation, scientific citation, Wikipedia entries, and memes in social networks [32,33,34,35];
- The distributions are stable over time;
- The popularity distributions for the major categories will be similar to each other and to the global one;
- The number of categories will grow more slowly compared to the number of games and players.

## 2. Materials and Methods

In our investigation about video game popularity, we employed data from a digital platform, *Steam*. This platform is a digital media distribution developed by Valve Corporation and an online game store responsible for managing the copyrights of the games in its database [36]. *Steam* currently has more than ninety million users [37], reaching more than twenty million users simultaneously online [38]. Unfortunately, due to user anonymity, we cannot determine users’ countries and, therefore, we are unable to provide player population by country. Nevertheless, the data is a representative sample of the global player population because network usage data by country is made available and one can see that there is network usage in all five continents, with China and the United States having the largest shares. It should also be noted that *Steam* was already the largest digital distribution platform for PC gaming in 2013 [39] in terms of users. Today, in *Steam*, there are more than fifty thousand video games [40].

Regarding user data, they are anonymous and unidentifiable. For example, we do not know who is playing, we only have information on how many users are playing each game in each hour. Eventually, the same user can play more than one game at a time. For example, if a user is playing two games simultaneously, he or she will be counted as a separate user in each of the two games. From the number of users per game per hour, one can obtain derived data such as the average number of users per hour in each month, as presented in the *steamcharts* website [41]. Because our analysis is limited to games indexed in *steamcharts*, we considered approximately twenty-two thousand games. Since the *Steam* data used here are not reported by the users, a reduction in subjective aspects of the analysis can be expected. Another type of data employed in our analysis is the meta information of each game (its release date, categories, etc.) on *Steam* [36]. From these data, we obtained the number of games released each month and the number of distinct games per category over time.

All data described above correspond to the period from July 2012 to December 2020, where games released prior to 2012 and games without time series were excluded. For user data per game per hour, *Steam* freely provides only from the current hour. Therefore, for creating a larger database, there needs to be a systematic accumulation of data over a given period. Although the *Steam* platform started in September 2003, this data storage process has only been freely made available by *steamcharts* from 2012 to the present day, justifying the starting date for the data used in our study. In turn, the data were downloaded and analyzed using Python scripts and the *Steam* API. These data are provided in .csv format as a dataset and can also be obtained in https://gitlab.com/tdfb/steam-data-2020/ (accessed on 16 June 2022). We note that the data collection method complied with the terms and conditions of the website. In addition, the sharing of the data also complies with the terms and conditions. To summarize, an outline of the data employed in this work is given in Table 1, where $\rho_{global}$ is the sum of the average concurrent players for all games and all months, while $\rho_{Indie}$ is the same sum but only for games in the largest category, *Indie*.

## 3. Results

**Figure 1:**
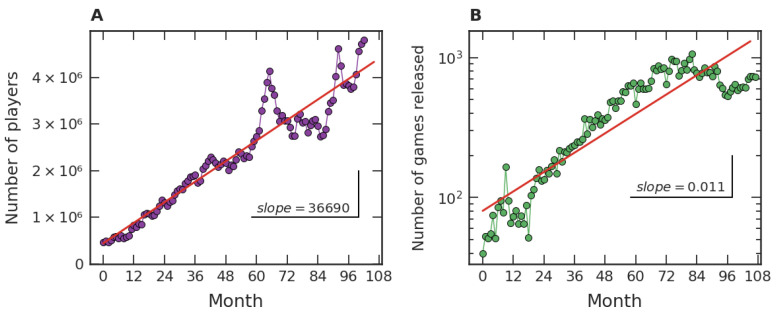
Number of players in and of games released on *Steam*. (**A**) The average number of players by hour in each month (discs) and a linear adjustment (straight line) along 111 months, where the first one is July 2012 and the last one is December 2020. (**B**) During these same months, growth of the number of games (discs) released and an exponential fit (straight line).

Our investigation on the popularity of video games initially focused on fairly general features of the dataset, which are exhibited in Figure 1. Figure 1A represents the time evolution of the average number of players per hour in each month in *Steam*. In an hour of December 2019, for instance, a few million users connected to *Steam* were in-game. Figure 1B presents the number of games released monthly in *Steam*, e.g., seven hundred and twenty-seven games were released in our dataset in December 2020. The data range from July 2012 to December 2020 and are organized in such a way that the first month (Month 1) represents July 2012, the second month (Month 2) represents August 2012, and so on. From Figure 1A we can note a mean linear growth tendency, with a growth rate of approximately thirty-seven thousand players per hour per month. On the other hand, Figure 1B shows a crude exponential growth trend of the games released on *Steam*, with an intrinsic growth rate close to 0.01/month.

**Figure 2:**
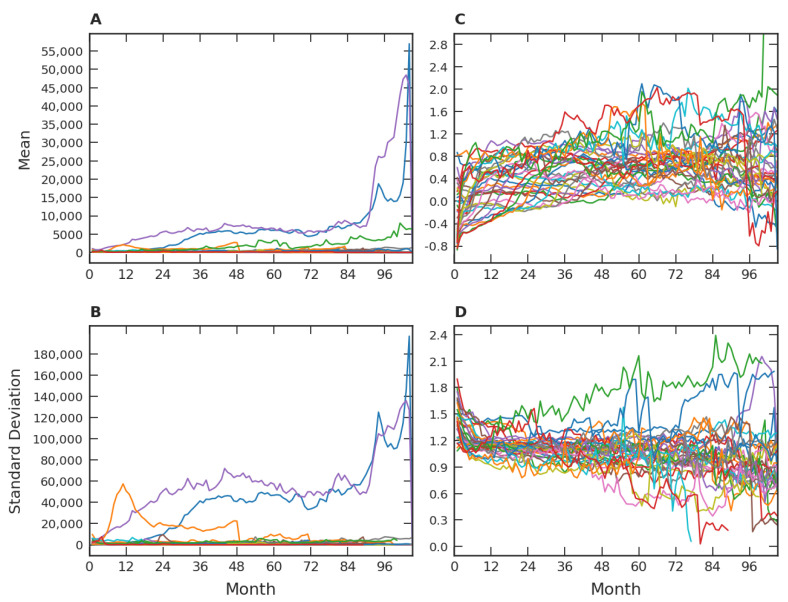
Mean and standard deviation of the trimester groups. (**A**) Mean of the average number of players per hour in each month ($x_{i}$) of all games of each trimester group along the months after release. (**B**) For these groups, the time evolution of their standard deviations. By using the logarithmic variable ($y_{i}=\log x_{i}$), the time dependence of the corresponding means (**C**) and standard deviations (**D**). Each one of the thirty-four trimester groups has its own color.

One way of further investigating video game popularity is made possible by dividing the data by release trimester (e.g., games released in January, February, or March of the same year would belong to one set). This data collation criterion coincides with the game releases by seasons. These sampling periods also correspond with quarterly reports to investors and shareholders. If we had chosen a monthly grouping window, we would have few games per window, especially in the first months of our database. For each trimester dataset, we analyzed the temporal evolution of popularity aspects in the subsequent months. Figure 2A,B show the time evolution of the means and standard deviations, where each color corresponds to a distinct trimester grouping. We used month one as the first month of each group and, consequently, the most recently launched sets of games have shorter time series, with the smallest series having two months. Due to outliers, the average behavior of different groups vary wildly in all ranges, clearly exemplified by the three upper curves in Figure 1A. A similar pattern is also observed with the standard deviation, only on a different scale. Note that the presence of such outliers makes it difficult to identify a general pattern in a limited range for all means and standard deviations.

A procedure that can favor the identification of a universal pattern related to Figure 2A,B come from attenuating the effects caused by outliers. In this direction, we will employ a new variable that smoothes outlier contributions. It is defined by:

(1) $$y_{i}=\log x_{i}\;,$$

where $x_{i}$ represents the average number of players by the hour in each month in the *i*-th game and log refers to the logarithm in base *e*. Using this new variable, we in fact verified an improvement in the temporal behavior of the means and the standard deviations, in the sense of revealing a more constant behavior after the first months after the game’s release. This fact is illustrated in Figure 2C,D.

**Figure 3:**
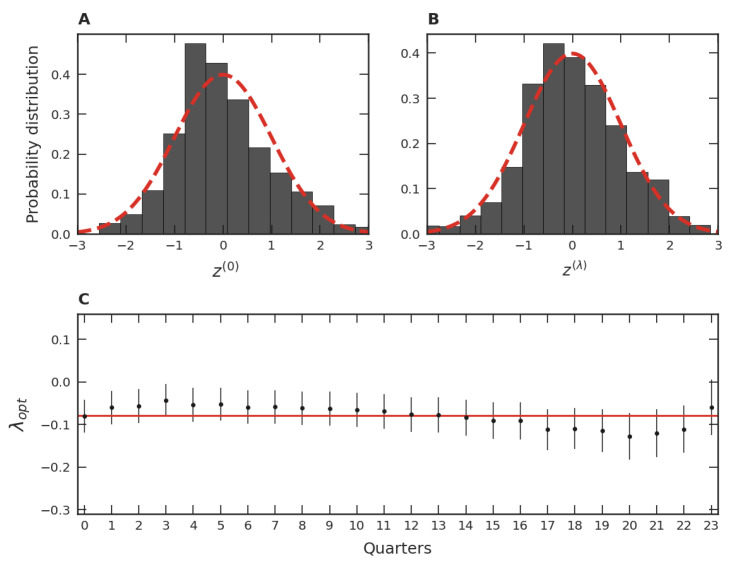
Box–Cox analysis of the 01/2015 trimester group. (**A**) Probability distribution function of the normalized logarithmic variable $z_{i}=\left(y_{i}-\langle y_{i}\rangle \right)/\sigma_{y}$ with $y_{i}=\log x_{i}$ for the 01/2015 trimester group, where $x_{i}$ is the average number of users per hour in each month of the *i*-th game. (**B**) The same as (**A**) but employing the optimal λ value ($\lambda_{opt}=-0.07$) of the Box–Cox transformation, Equation (3). The dashed lines refer to a normal distribution with the mean zero and unity standard deviation. (**C**) Time evolution of the optimal parameter λ, $\lambda_{opt}$, of the trimester group along the twenty-four quarters. The discs represent the $\lambda_{opt}$’s along the time evolution of the 01/2015 trimester group and the straight line of the mean of all quarters, $\langle \lambda_{opt}\rangle =-0.08$, which is close to the value obtained with the $\lambda_{opt}$ obtained via the maximum-likelihood method in (**B**). The error bars refer to the 95% confidence interval of the $\lambda_{opt}$’s estimated via the maximum-likelihood method.

To go beyond means and standard deviations, we move our study in the direction of probability distributions. This approach enables us to investigate relative popularity among video games, since the relative frequency of occurrences of video games with a different number of players can be put in evidence. With this goal in mind, we firstly determined probability density functions (PDFs) of $y_{i}$. Note that, due to the approximate constancy of each mean and standard deviation previously discussed, PDFs that trend towards stability are expected. Pursuing the investigation of these PDFs, we grouped the games in trimesters by their release date, as employed in the discussion of means and standard deviations. To conduct an exploratory analysis of the data, we initially chose to investigate one of these release date trimesters separately. The group randomly chosen from the thirty-four trimesters in our database was the one with games released in the first trimester of 2015 (01/2015). Motivated by the approximate constancy of means and standard deviations (Figure 2C,D), we grouped the monthly average of players per game per hour in each subsequent quarter until December 2020, giving 24 quarters of time evolution. Since there are 420 games in the 01/2015 trimester, the quantity of $y_{i}$ considered is 420 × 24 (=10,080). Figure 3A shows the PDF of these 10,080 data, where we used the normalized variable *z*, that is,

(2) $$z_{i}=\frac{y_{i}-\langle y_{i}\rangle }{\sigma_{y}},$$

with $\langle y_{i}\rangle$ and $\sigma_{y}$ being respectively the mean and standard deviation of the quarterly set of $y_{i}$’s. As we can see from this figure, the PDF is somewhat left-skewed. Proceeding similarly, we verified that this slightly asymmetrical pattern occurs in the other remaining thirty-three release date trimesters of our database.

As can be seen, the graph of the data in Figure 3A resembles a Gaussian in the variable *z*, that is, a lognormal distribution in the variable *x*. Despite this similarity, the slight asymmetry of the data distribution, when compared to the normal one of mean zero and unit standard deviation, suggests a necessity of a more fine-grained variable transformation than the logarithm. In cases like this, the Box–Cox transformation is usually employed [42,43,44,45,46]:

(3) $${y_{i}}^{\left(\lambda \right)}=\left\{\begin{array}{c} \frac{{x_{i}}^{\lambda }-1}{\lambda }\;\;\;\;\;\left(\mathrm{if}\;\lambda \neq 0\right)\\ \log x_{i}\;\;\;\;\left(\mathrm{if}\;\lambda =0\right) \end{array}\right.,$$

where, at the limit λ→0, the logarithmic behavior is recovered and the value of the parameter λ indicates the degree of deviation from this behavior.

A standard procedure to arrive at an optimal λ is the maximum likelihood method [47]. The result of this procedure, applied to the same data of Figure 3A, can be seen in Figure 3B. In this figure, we utilized the normalized variable:

(4) $${z_{i}}^{\left(\lambda \right)}=\frac{{y_{i}}^{\left(\lambda \right)}-\langle y_{i}^{\left(\lambda \right)}\rangle }{\sigma_{y^{\left(\lambda \right)}}},$$

where $\langle y_{i}^{\left(\lambda \right)}\rangle$ is the mean value μ of the more than ten thousand $y_{i}^{\left(\lambda \right)}$’s and $\sigma_{y^{\left(\lambda \right)}}$ is the corresponding standard deviation σ. It is noticeable that the degree of symmetry of the data distribution in Figure 3B is greater than that in Figure 3A. This gain in symmetry was verified for all the thirty-four quarterly groups of our database. Note also that, in terms of the original variable and employing the Box–Cox transformation, the popularity distribution is given by:

(5) $$p\left(x\right)=\frac{1}{\sqrt{2\pi \sigma^{2}}}\frac{1}{x^{1-\lambda }}\exp\left[-\frac{{\left(\frac{x^{\lambda }-1}{\lambda }-\mu \right)}^{2}}{2\;\sigma^{2}}\right],$$

where x≥0 and $\left(x^{\lambda }-1\right)/\lambda$ is replaced by logx when λ=0, reducing p(x) to a lognormal distribution.

Despite our grouping of the twenty-four quarters of the time evolution for the 01/2015 trimester data, it should be noted that we can apply the Box–Cox transformation separately for each of these quarters. The results of these applications are PDFs comparable to the one presented in Figure 3B. In addition, the optimal λ for each one of these PDFs is close to the $\lambda_{opt}$ considered in Figure 3B, as presented in Figure 3C. Similar results were verified for each optimal λ value of the other thirty-three trimesters of our data. These results indicate that the PDFs of each one of the thirty-four trimester groups are both stable and similar to each other over time, justifying the use of the grouping process employed in Figure 3A,B.

**Figure 4:**
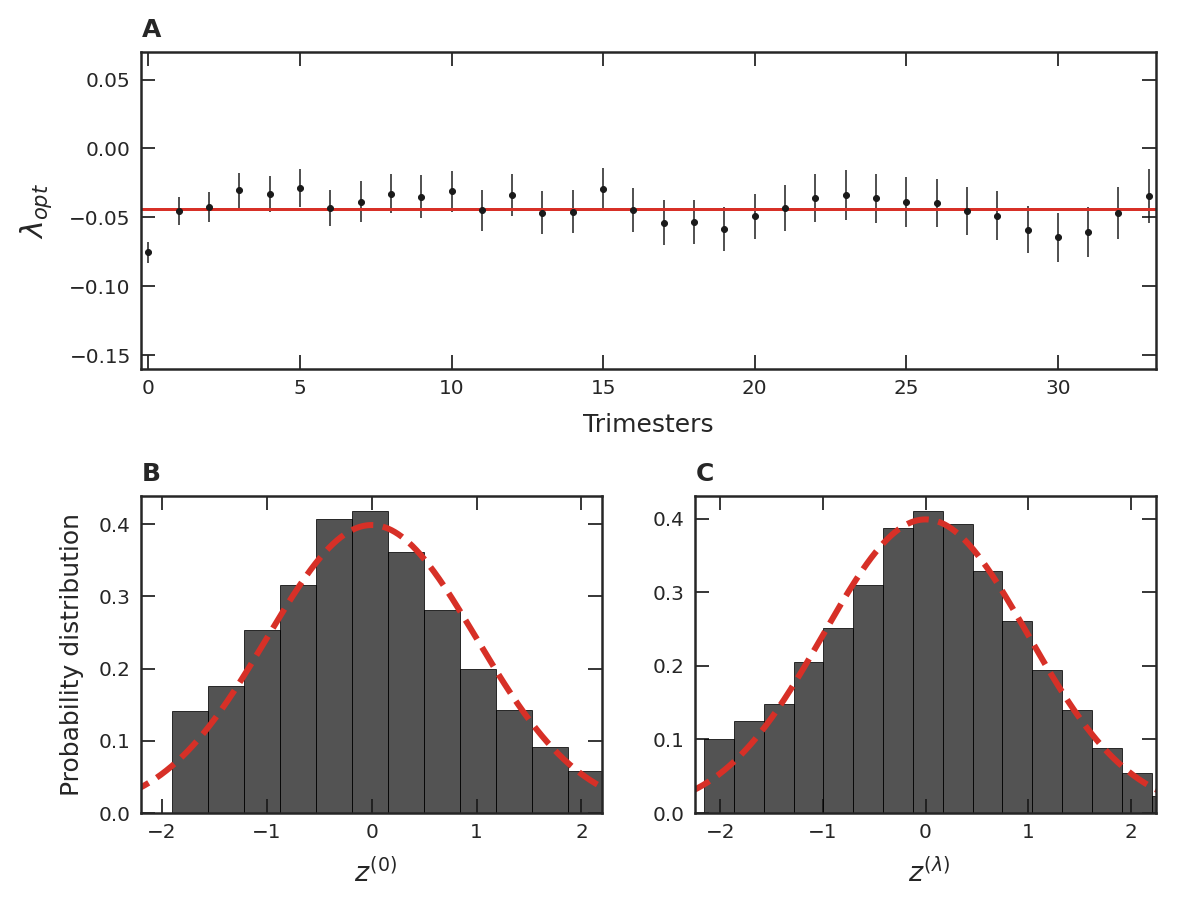
Box–Cox analysis for all trimester groups. (**A**) Optimal λ, $\lambda_{opt}$, for each trimester group (disc) and the mean value, $\bar{\lambda_{opt}}=-0.04$, represented by the red straight line. The error bars indicate 95% confidence intervals. (**B**) For the normalized global data, the PDF of the logarithmic variable, $z^{\left(0\right)}$. (**C**) The same as in (**B**) but employing $\lambda_{opt}=-0.05$ in the Box–Cox transformation. The dashed lines correspond to the normal distribution with mean zero and unity standard deviation.

When a procedure analogous to that discussed in connection to Figure 3B is applied to each one of the thirty-four trimester groups, a mean $\lambda_{opt}$, $\bar{\lambda_{opt}}$, is obtained. These values of $\lambda_{opt}$ are shown in Figure 4A. Among all $\lambda_{opt}$’s, the smallest $\lambda_{opt}$ is −0.07, the largest is −0.02, and the mean is −0.04. These facts point to a similarity of the PDFs of the trimester data normalized via the transformation given in Equation (4). This robustness also suggests investigating the behavior of all standardized trimester data as a unique data set via a single PDF. In this case, Figure 4B illustrates how the distribution in terms of the variable $z^{\left(0\right)}$ would be. Note that the slight asymmetry of this data distribution is consistent with that of the dataset presented in Figure 3A. In turn, when the variable $z^{\left(\lambda \right)}$ with the maximum likelihood is employed, we obtain the optimal global parameter equal to $\lambda_{opt}=-0.05$. Note that this value is close to the behavior of the different $\lambda_{opt}$’s shown as dots in Figure 4A and also close to the mean value of all $\lambda_{opt}$’s, shown as the continuous line. From Figure 4B,C, we also verify that the similarity with the Gaussian is accentuated when $\lambda_{opt}$ is used instead of λ=0, just as it was for the data used in Figure 3A,B. These facts point to a unifying standard PDF of the normalized variable, including all thirty-four trimesters as well as their time evolution.

**Figure 5:**
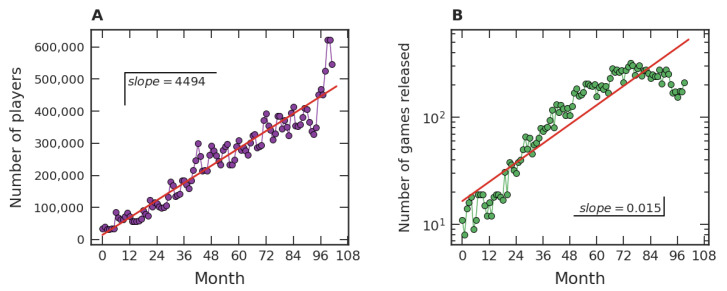
Number of players in and of games released on *Steam* for the *Indie* category. (**A**) The average number of players per hour in each month for the *Indie* category (purple discs) and a linear adjustment (straight line, red), where month one is July 2012 and the last month is December 2020. (**B**) During these same months, growth of the number of games released in this category (green discs) and an exponential fit (straight line, red).

Another way of focusing on the popularity of video games involves comparing games with others of the same category (also called *tag* in *Steam*). The category of a video game is indicated by developers or users. In our analysis, we use the user-defined categories (decided by popular vote) as the classification of a game. The twelve categories with the largest number of games are *Indie* (15,348), Casual (9201), Action (8705), Adventure (8601), Single Player (6835), Simulation (5514), Strategy (5063), RPG (4027), 2D (3451), Puzzle (2753), Atmospheric (2479), and Early Access (2351), in which the numbers in parentheses indicate the number of games as of December 2020. Note that there is overlap between the categories, e.g., a game could belong to both the *Indie* and to the Puzzle category. In Figure 5A, the time evolution of the average number of players per hour in each month from July 2012 to December 2020 of the largest category, *Indie*, is shown. As can be seen, there is a mean linear growth trend similar to the one identified in the general case shown in Figure 1A. Linear growth trends over time of the average number of players per hour have also been verified for the majority of the twelve largest categories. Other categories, which have less than 150 games, show many statistical fluctuations in their temporal evolution, making it difficult in most cases to identify a pattern. On the other hand, as in the general case (Figure 1B), the number of games released monthly exhibits an approximate exponential growth trend (at least for the intermediate months) for most of the twelve major categories of games. This behavior is illustrated for the *Indie* case in Figure 5B. For each of the other categories of games, the number of games released per month is smaller and the possible identification of an exponential behavior becomes less clear.

**Figure 6:**
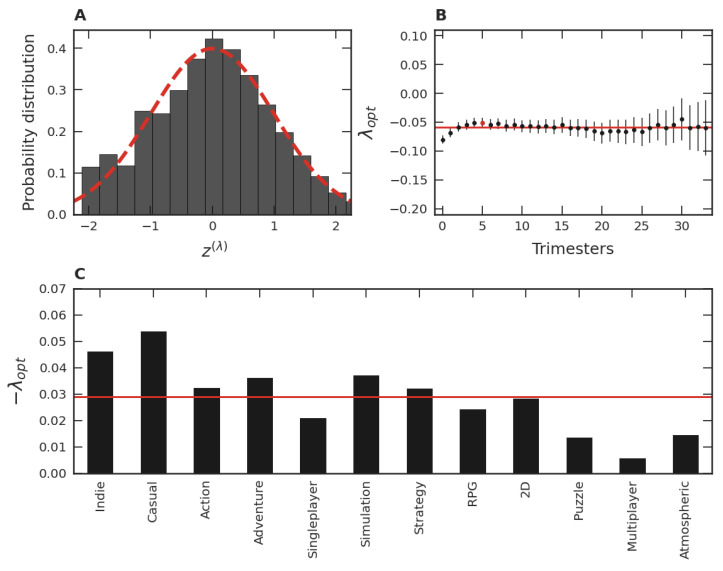
Video game categories. (**A**) PDF for the *Indie* category obtained via the Box–Cox transformation ($\lambda_{opt}=-0.049$), analogous to Figure 4C (all quarters of all trimester groups together). (**B**) The Box–Cox parameter for *Indie* games in each trimester of our dataset, analogous to Figure 4A. The bars refer to 95% confidence intervals. The straight line refers to the mean of all trimestral Box–Cox parameters, $\langle \lambda_{opt}\rangle =-0.06$. (**C**) Optimal Box–Cox parameter, $\lambda_{opt}$, for the twelve largest game categories, calculated via the same procedure of (**A**). The $\lambda_{opt}$’s are sorted in descending order of their number of games by category. The straight line represents the average of these $\lambda_{opt}$’s, 〈λ〉=−0.028 for the twelve major categories.

Continuing with the analysis of the popularity of categorized video games, Figure 6A is analogous to Figure 4C (all quarters of all trimester groups together) focusing on the distribution of the number of users for *Indie* video games. In this case, one has a Box–Cox transformation with $\lambda_{opt}=-0.049$. This value is consistent with the $\lambda_{opt}$ obtained for each quarterly data of the *Indie* category, shown in Figure 6B (the analogous of Figure 4A). Similar behaviors to those shown in Figure 6A,B were also obtained for the other eleven major categories of games, whose $\lambda_{opt}$’s are presented in Figure 6C. In turn, the mean of these twelve average values of $\lambda_{opt}$ is equal to −0.028, a result close to the global mean shown in Figure 4B. The calculation of these optimal λ’s for the remaining categories is also close to −0.03 in most cases, even though the number of games involved in each category is small.

**Figure 7:**
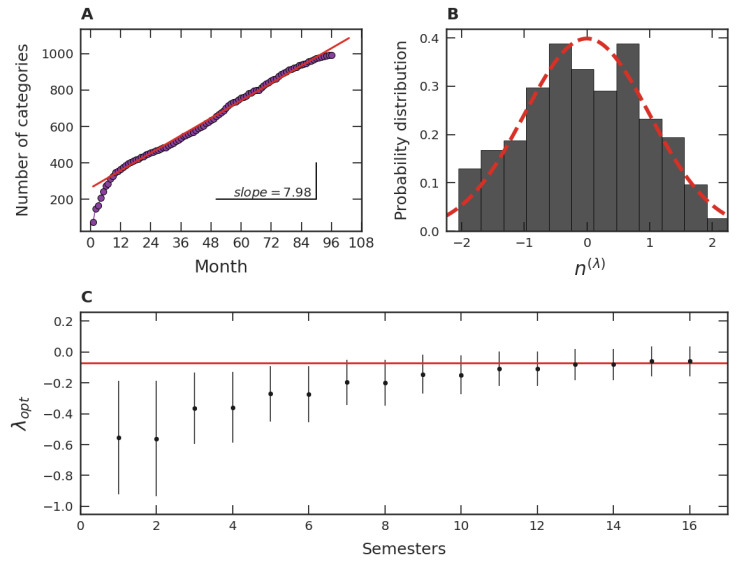
Distribution of video game categories. (**A**) Time evolution of the number of categories along the months from July 2012 to December 2020. The straight red line represents a linear adjustment from month 10 to the last one. (**B**) PDF for categories obtained via the Box–Cox transformation ($\lambda_{opt}=-0.12$) as a function of the number of video games in each category in December 2020. The dashed line refers to a Gaussian distribution of mean zero and unity standard deviation. (**C**) Time evolution from 2012 to 2020 of the $\lambda_{opt}$, where each disc represents a semester. The bars indicate 95% confidence intervals of $\lambda_{opt}$ parameters. The straight red line exhibits the mean behavior of the last two years.

As the last stratum of our analysis, we examine further aspects of the popularity of video game categories. The first concern considered was the growth of the number of distinct video game categories over time. As shown in Figure 7A, we identified a linear growth trend, whose increase rate is approximately 8 categories/month. In turn, the approach to investigate the relative popularity among categories is similar to the one of Figure 6A,B. In this investigation, we focus on the relative popularity distribution of categories as a function of their number of video games. By using an optimal Box–Cox transformation, this distribution in December 2020 is displayed in Figure 7B. In December 2020 there were 1044 categories, encompassing 21,752 games. Despite having many categories with only one game, we did not consider them. This is because the naming of the pertinent category can be quite uncertain since there is a very limited number of votes for these categories, which leads to statistical uncertainties. As we can see from Figure 7B, the Box–Cox transformed data can be adjusted by a normal distribution of mean zero and unity standard deviation, but in a less precise manner than in our previous studies (Figure 3B, Figure 4C and Figure 6A). However, the optimal λ, $\lambda_{opt}=-0.12$, is close to the ones previously found, indicating a unified view for our study of the popularity of video games. This result is reinforced by the stability of $\lambda_{opt}$ time evolution in recent years, shown in Figure 7C. In the early years of our database, there were both few categories and few games, worsening the adjustment of the data by a Box–Cox distribution and leading to an unclear $\lambda_{opt}$ value, evidenced by wider confidence intervals. As in previous analyses, since $\lambda_{opt}$ is close to zero, the data as in Figure 7B can be represented by a lognormal distribution in a first approximation.

To summarize our quantitative results about the comparative popularity of video games, we present in Table 2 typical values of optimal λ as well as the corresponding Box–Cox transformed mean (μ), standard deviation (σ), skewness (γ), and kurtosis (κ) for the popularity distribution of video games both globally and per-category. If the PDF, considering a convenient variable transformation, was Gaussian, it would be symmetric in relation to μ, γ=0, and there would be a balance between the distribution tail and its peak, κ=0 [48]. Therefore, deviations from these values indicate discrepancies from normality. For instance, if a logarithmic variable (λ=0) is used and if γ and κ are close to zero, these values of γ and κ indicate, in a first approximation, that the data can be seen as lognormally distributed (usually, this approximation could be improved by employing an optimal λ). In terms of the original variables, the optimized popularity distribution for each stratum presented in Table 2 is obtained using the corresponding values for λ, μ, and σ in the Equation (5).

## 4. Discussion

Our empirical results about the popularity of video games, based on one of the major gaming platforms (*Steam*), suggest a robust scenario. Firstly, our studies are consistent with the notion of a crescent number of video game players around the world, as well as with an increase in video games released over time. Quantitatively, the average number of players per hour along the months approximately displays a linear growth, which is manifested both from a global point of view (Figure 1A) and from the perspective of game categories (Figure 5A), especially for the major ones. For the number of distinct categories, a pattern of growth similar to the ones presented in Figure 1B and Figure 5B was also verified in Figure 7A. As for the evolution of the number of released games, an approximately exponential growth trend was identified at the global level (Figure 1B). This behavior of linear (arithmetic) resource growth and exponential (geometric) demand has already been well explored by Malthus [49]. Indeed, players will occupy the role of resources and games and the role of demand in the Malthusian view if we interpret games as competing with each other for players’ attention. In this case, the exponential increase drastically dominates the linear one in the long term. In addition to Figure 1A,B, a Malthusian pattern of growth is also present when analyzing the data in categories (Figure 5A,B), but less robust. These studies point to common patterns of growth in the three levels under investigation: global data, major categories, and among categories. The Malthusian behavior is common in population dynamics in biological [50] and social [51] systems when there are no limitations, typically occurring in the early stages. On the other hand, restrictions to this initial growth regime commonly manifest over time, indicating some kind of population saturation [52].

Another aspect investigated in this work was the relative popularity of the games, in this case, made through probability distributions. In our results, we conducted the lognormal distribution by considering the stability of log-transformed means and standard deviations (Figure 2). However, for the purposes of this discussion, we emphasize another viewpoint. In this direction and from a qualitative perspective, one could consider that each video game release fragments (partitions) of the player base in a given proportion. In turn, successive releases lead to subsequent fragmentation (partition) of the player base. The simplest hypothesis is that the games are unknown to the players at the time of their release and, as a consequence, one could assume in a first approximation that these successive fragmentations occur entirely at random and independently of each other, that is, this multiplicative process occurs in a memoryless manner. This simple hypothesis is an essential condition for obtaining the lognormal distribution [53]. An important result of this study was to detect similar approximate lognormal patterns across three different strata. Despite the apparent simplicity of the memoryless (randomness) hypothesis, the lognormal distribution has been successfully employed in the most diverse contexts, such as in the investigation of news and memes [35,54], proportional elections [55], or in the network analysis of seed dispersion [45].

As discussed above, we found that the lognormal distribution is a first approximation of the empirical distribution. Deviations from this distribution are related to the violation of the simple hypothesis; that is, some factors we assume to drive the lognormal distribution are not fully true. It is unreasonable to assume that games are completely unknown to players at the time of their release, or that ignorance remains over time. For instance, each game has its defining characteristics, its peculiarities, its target audience, and its business model. Moreover, there exists some cultural memory due to advertisements and well-established game franchises. Communicative memory between players, such as social networks, anticipation for a game’s release, and the accumulation of fame, also change the perception of games. Since all these factors disturb the memoryless (randomness) hypothesis, they can contribute to explaining the asymmetry shown by Figure 3A and Figure 4B. Quantitatively, the $\lambda_{opt}$ parameter provides a measure of the departure from the initial hypothesis. Thus, we interpret the magnitude of $\lambda_{opt}$ as a measure of the presence of cultural and communicative memories in the distribution of popularity, where $\lambda_{opt}=0$ corresponds to the memoryless hypothesis. This finding is consistent over time (Figure 3C), across different game groups (Figure 4A), and in the game categories (Figure 6B). Despite small variations around the average (showing that the popularity of some games is more affected by memories than others), the effect of memories is present in all strata investigated.

Some particularities of the game categories can be concretely related to cultural and communicative memories. As Figure 6C shows, some categories have the $\lambda_{opt}$ magnitude notably larger than others. Particularly, the popularity of the *Indie* and Casual categories are most affected by memories. An explanation consistent with our interpretation is that this is due to the business model adopted by the developers of these game categories. For instance, *Indie* games are considered independent, relatively small productions in the gaming market. Frequently, these games are unknown to the public, and the goal of these small developers is to sell as many games as possible at a fair price. In this way, the games that survive and achieve success are through word of mouth among players, characterizing a high communicative memory. In the same vein, Casual games have similar features, that is, they do not usually involve expensive productions, are considered a hobby of a few hours, and their business model is similar to that of *Indie*. In contrast, the business model adopted by Multiplayer game developers is quite different. Most of these games are “freemium”, meaning they are free or inexpensive, where the goal is to keep people playing as much as possible and spending money on customization. Since these games are readily available to every user, the decision to play or not is entirely up to the person, favoring the hypothesis of randomness and, consequently, the small magnitude of $\lambda_{opt}$. Despite variations, all $\lambda_{opt}$’s found were negative. The more negative the $\lambda_{opt}$, the more asymmetric is the empirical distribution (see Figure 3A and Figure 4B), favoring the presence of outliers. Thus, memory mechanisms seem to contribute to increasing the popularity inequality among video games by promoting the existence of highly popular games. This inequality was further investigated regarding the distribution of video game categories (Figure 7B) over time (Figure 7C). Curiously, the patterns discovered were consistent with the ones found in Figure 3B and Figure 4C, and in Figure 3C and Figure 4A, respectively. A possible reason for this resemblance could be that some video game developers try to develop games belonging to popular categories, since it is difficult to gauge popular reception of an unknown genre and, therefore, there is a greater risk of commercial failure.

For the objectives outlined in this work, the Box–Cox transformation was sufficient to identify memory effects and to describe prominent aspects of the relative popularity distributions. Without doubt, other families of distributions could be employed in the direction of further refining the empirical data description. In particular, a limitation of the present study is that we are unable to clearly separate cultural and communicative memories and, thus, our interpretations of the magnitude of the λ parameter rely on domain knowledge. As a next step in improving the model, one could, in a direction similar to the one proposed in Reference [3], try to untangle the memory phenomena via a model with extra parameters (for instance, a multivariate Box–Cox transformation could potentially be employed, where each of the parameters would be interpreted as a distinct memory mechanism). However, this new hypothetical model would probably require other types of data to correctly quantify the diverse memory mechanisms (e.g., the number of unique user *tweets* that refers to a game in the weeks following its release as a way to gauge communicative memory). In addition, it would be worthwhile to re-investigate the linear growth of the number of players and the exponential number of games released to amplify the coverage of our analysis and to confirm if the Malthusian competition remains valid. This latter point would be of special value if it were possible to combine several game databases since, if the growth trends are similar across different bases, this could indicate the existence of a universal video game consumption pattern.

Finally, we believe that the model proposed to describe the relative popularity could potentially contribute to psychological and sociological investigations. For instance, a perennial issue both in academic [19,56] and quotidian [57] discussions is if video game usage contributes to violent behavior. A possible future application of the work presented here would be, first, to correlate the popularity of violent game categories (e.g., *Steam* has games with the Gore and Violent tags) with socioeconomic indicators in the same period. If the relative popularity of these categories is better described by a Box–Cox transformed distribution rather than by a lognormal, this could be suggestive of a communicative memory effect; that is, a collective, user-centered preference for violent games. Such description could then be utilized to better understand this consumption pattern and to serve to support the psychological/social sciences in formulating hypotheses to answer the aforementioned issue.

## Figures and Tables

**Table 1:** A summary of the data utilized in this work.

Total of games: 21,752	Number of *tags*: 1044	
Δt: 8.5 years	$t_{i}$: July 2012	$t_{f}$: December 2020
$\rho_{global}$: $1.78\times 10^{8}$	$\rho_{Indie}$: $2.46\times 10^{7}$	

**Table 2:** Statistical parameters for the transformed data. The parameters of the Box–Cox transformed data (first column): Box–Cox parameter (λ), mean (μ), standard deviation (σ), skewness (γ), and kurtosis (κ). Values for these parameters are shown for log-transformed (second column), Box–Cox all data transformed (third column), Box–Cox transformation for the *Indie* category (tag) (fourth column), and Box–Cox transformation for the distribution of the number of video games by category (fifth column). After the ± symbol, the 95% confidence interval of each parameter are exhibited.

	Lognormal (All Data)	Box–Cox (All Data)	Box–Cox (*Indie* Tag)	Box–Cox (Games by Tags)
λ	−0.00	−0.053±0.001	−0.049±0.002	−0.12±0.05
μ	−0.27±0.01	−0.43±0.02	−1.60±0.02	−3.72±0.25
σ	−1.2±0.1	−2.6±0.2	−2.6±0.2	−0.74±0.15
γ	−0.41±0.02	−0.01±0.01	−0.01±0.01	−0.03±0.05
κ	−0.15±0.07	−0.28±0.02	−0.34±0.03	−0.5±0.6

## Data Availability

Data was obtained from *Steamcharts*, *SteamDB*, and *Steam*, and are available at https://steamcharts.com/ (accessed on 10 January 2021), https://steamdb.info/ (accessed on 10 January 2021) and https://store.steampowered.com/search/?term= (accessed on 15 January 2021), respectively. A Gitlab repository with the dataset employed in this work is also available at https://gitlab.com/tdfb/steam-data-2020 (accessed on 16 June 2022), containing the data in .csv and .pkl format, a description of data curation procedures, and a guide to re-obtain the results presented in the article.
